# Long‐Term Performance and Bulk‐Isotope Effects in Methane Clumped‐Isotope Measurements Using a 253 Ultra HR‐IRMS

**DOI:** 10.1002/rcm.70198

**Published:** 2026-10-23

**Authors:** Naizhong Zhang, Alexis Gilbert, Mayuko Nakagawa, Jakub Surma, Jan G. C. Meissner, Joachim Mohn, Stefano M. Bernasconi, Yuichiro Ueno, Naohiro Yoshida

**Affiliations:** ^1^ Department of Earth and Planetary Sciences Institute of Science Tokyo Tokyo Japan; ^2^ Laboratory for Air Pollution/Environmental Technology Empa Dübendorf Switzerland; ^3^ Institute for Marine and Atmospheric research Utrecht (IMAU) Utrecht University Utrecht the Netherlands; ^4^ Department of Earth and Planetary Sciences ETH Zurich Zürich Switzerland; ^5^ Department of Earth, Atmospheric and Planetary Sciences (EAPS) Massachusetts Institute of Technology Cambridge Massachusetts USA; ^6^ Earth‐Life Science Institute Institute of Science Tokyo Tokyo Japan

## Abstract

**Rationale:**

Changes in instrument condition over time may affect methane clumped‐isotope measurements, requiring long‐term monitoring of calibration performance. Whether the 253 Ultra calibration framework remains valid over multiyear operation and applicable across natural bulk‐isotope compositions, particularly to low‐δD methane with weak ^12^CH_2_D_2_ signals, has not been systematically evaluated.

**Methods:**

Long‐term performance of the Science Tokyo 253 Ultra was evaluated from 2019 to 2026 using reference‐gas zero tests, repeated working‐gas measurements, and independently prepared equilibrated methane gases. Equilibrated gases with different δ^13^C and δD compositions were used to test for possible bulk‐isotope dependence in Δ^13^CH₃D and Δ^12^CH_2_D_2_ measurements. External consistency was assessed using nine shared methane gases measured across seven laboratories with 253 Ultra HR‐IRMS, Panorama HR‐IRMS, and mid‐infrared laser spectroscopy.

**Results:**

Reference and working gases showed no temporal drift, and equilibrated gases measured from 2021 to 2026 remained consistent with the calibration framework established in 2020. No systematic dependence of Δ^13^CH₃D or Δ ^12^CH_2_D_2_ on either δ^13^C or δD was resolved, including for equilibrium gases with δD values as low as −335‰. Science Tokyo measurements were generally consistent with results from other laboratories across a wide range of bulk‐isotope compositions and nonequilibrium clumped‐isotope signatures.

**Conclusions:**

The calibration framework established for methane clumped‐isotope measurements with the Science Tokyo 253 Ultra HR‐IRMS remained stable and applicable over multiple analytical campaigns. Validation measurements and interlaboratory comparisons demonstrated consistent results across a broad range of methane bulk‐isotope compositions, without the need for campaign‐specific or additional bulk‐isotope–dependent corrections, with δD values extending down to −335‰.

## 1 Introduction

Conventional bulk isotopic compositions of methane, δ^13^C and δD values, have long been used to distinguish sources and infer formation pathways. However, their interpretation is often nonunique because bulk isotopic signatures are influenced by substrate compositions, formation mechanisms, and secondary processes such as mixing, diffusion, and oxidation. The combination of these factors can result in similar or overlapping bulk isotopic signatures [1]. Over the past decade, methane clumped‐isotope analysis, based on the abundances of the two doubly substituted isotopologues ^13^CH₃D and ^12^CH_2_D_2_, has become a powerful approach that provides additional constraints on methane formation and alteration processes [2, 3, 4, 5, 6, 7, 8, 9, 10]. Unlike bulk isotope signatures, which are influenced by isotope exchange between methane and external carbon and hydrogen pools, clumped‐isotope signatures reflect the distribution of isotopes within individual methane molecules and their approach to or departure from internal isotopic equilibrium. In principle, the equilibrium distributions of methane clumped isotopologues are primarily controlled by temperature [3]. In many natural systems, however, methane may deviate from isotopic equilibrium because of the kinetic isotope effect and combinatorial effect during methane formation, or postformation processes such as source mixing, diffusion, and microbial alteration [7]. These equilibrium and disequilibrated signatures can provide process‐specific constraints on methane formation and consumption pathways that are less directly dependent on substrate isotopic composition, offering information beyond that available from δ^13^C, δD, and methane concentration measurements alone.

To date, two major analytical approaches have been developed for methane clumped‐isotope measurements: high‐resolution isotope‐ratio mass spectrometry (HR‐IRMS) and mid‐infrared laser spectroscopy. HR‐IRMS measurements are primarily performed using the Thermo Scientific 253 Ultra [2, 8, 9, 11, 12, 13] or the Nu Panorama [6, 7, 14, 15], whereas laser‐based measurements have mainly employed quantum cascade laser absorption spectrometers (QCLAS) developed by Aerodyne Research [4, 5, 10]. Because these platforms are based on different physical principles, they are subject to different analytical artifacts and require platform‐specific correction and calibration procedures.

The 253 Ultra has been widely used to analyze CH₄ samples from both laboratory experiments and natural settings [9, 13, 16, 17, 18, 19, 20, 21, 22, 23], offering high analytical precision of approximately 0.3‰ for Δ^13^CH₃D and 1.5‰ for Δ^12^CH_2_D_2_, with relatively low sample requirements of 80–220 μmol CH₄ (approximately 2–5 mL at STP). Across current analytical platforms, methane gases equilibrated at known temperatures are generally used to establish the absolute Δ^13^CH₃D and Δ^12^CH_2_D_2_ scales. Laboratory reference gases (RGs) assigned values on these scales are then used to transfer and monitor the calibration during routine sample measurements. For the 253 Ultra, once such a calibration framework is established, it may be applied over several years of instrument operation, making both long‐term instrumental stability and the continued validity of the framework essential for reliable data interpretation.

Assessing this long‐term validity requires more than demonstrating short‐term analytical precision. Methane clumped‐isotope measurements require repeated optimization of ion‐source conditions, mass resolution, ion‐optical focusing, and collector positioning. Changes in these settings, together with changes in the condition or configuration of the narrow entrance slit, may alter signal transmission, peak position, and peak shape. These effects are particularly important for Δ^12^CH_2_D_2_ measurements, for which the low‐intensity ^12^CH_2_D_2_^+^ beam must be separated from the adjacent and substantially more intense ^13^CH₃D^+^ and ^13^CH₅^+^ peaks, with their residual contributions removed through a tailing correction based on an independently measured water‐peak shape [8, 9, 13]. Long‐term performance may also depend on the stability of the compact discrete dynode (CDD) ion‐counting response and background. Repeated instrumental tuning, maintenance, detector aging or replacement, and switching among different high‐resolution isotopologue applications, such as ^15^N^15^N measurements in N_2_ and D_2_ measurements in H_2_, could therefore introduce time‐dependent offsets and compromise the continued validity of the calibration framework.

A second question is whether calibrated Δ^13^CH₃D and Δ^12^CH_2_D_2_ values remain independent of bulk isotopic composition across the range encountered in natural samples. Although clumped‐isotope compositions are expressed relative to a stochastic distribution, variations in δ^13^C and δD alter the absolute abundances of ^13^CH₃D and ^12^CH_2_D_2_ and consequently their signal intensities. These changes in isotopologue abundance and signal intensity may alter the magnitude of the instrumental effects described above, and incomplete correction may introduce composition‐dependent biases into calibrated Δ values.

Bulk‐isotope–dependent effects have been reported for laser‐based methane clumped isotope measurements, although they arise from platform‐specific spectroscopic effects and correction procedures [5, 10]. The possible influence of bulk isotopic composition has also been discussed for the Panorama HR‐IRMS. Young et al. [6] evaluated potential effects associated with sample–reference D/H differences, including peak tailing and ion‐source scrambling, and concluded that these effects were smaller than the analytical precision under their typical operating conditions. Consistent with this assessment, their subsequent study did not report the need for an explicit bulk‐isotope–dependent correction [7]. In contrast, Liu et al. [15] identified significant δD‐related nonlinearity in both Δ^13^CH₃D and Δ^12^CH_2_D_2_ using another Panorama HR‐IRMS and applied composition‐dependent corrections. These differing observations indicate that such effects may depend on the individual instrument and its operating conditions and should therefore be evaluated independently for the 253 Ultra. A previous study using a 253 Ultra found no significant dependence of calibrated Δ^13^CH₃D or Δ^12^CH_2_D_2_ values on δD for equilibrated methane gases with δD values ranging from approximately −160‰ to +20‰, with the observed variations remaining within analytical precision [8]. However, whether this conclusion holds over the broader δD range encountered in microbial methane remains unclear.

This issue is particularly important for low‐δD microbial methane, which can extend to −400‰ or lower [24]. Because the stochastic abundance of ^12^CH_2_D_2_ is approximately proportional to the square of D/H, low‐δD methane produces substantially smaller ^12^CH_2_D_2_ ion beams than methane with higher D/H ratios. Working RGs used in published 253 Ultra methods generally have thermogenic‐like δD values of approximately −180‰ to −150‰ [8, 9, 11, 12, 13]. Measurements of thermogenic methane therefore involve relatively modest sample–reference differences in ^12^CH_2_D_2_ signal intensity, whereas low‐δD microbial methane produces a much larger signal mismatch. Under these conditions, residual contributions from neighboring peak tails or instrumental background constitute a larger fraction of the sample signal, placing greater demands on the accuracy of peak‐tailing and background corrections. Lower count rates may also make the measurements more susceptible to subtle variations in ion‐counting response.

The 253 Ultra at Science Tokyo provides a unique multiyear record of methane clumped‐isotope measurements, comprising repeated zero tests, laboratory RG analyses, and independently prepared equilibrium gases. In this study, we use these data to evaluate the long‐term performance and stability of the Science Tokyo 253 Ultra, test whether the 2020 calibration framework remained applicable to equilibrated gases measured in subsequent years, and determine whether measured Δ^13^CH₃D and Δ^12^CH_2_D_2_ values show systematic dependence on bulk δ^13^C or δD. By extending this evaluation to equilibrated methane with δD values as low as −335‰, we also assess whether an additional correction for bulk‐isotope effects is required over this extended δD range.

## 2 Methods and Experiments

### 2.1 Methane Bulk and Clumped‐Isotope Analysis Using 253 Ultra

Methane bulk and clumped‐isotope compositions were measured using a Thermo Scientific 253 Ultra HR‐IRMS at Science Tokyo. The instrument was installed in 2018 and has been used for methane clumped‐isotope analysis since late 2019. The original calibration framework was established using equilibrated methane gases in 2020 [9]. During the study period, the instrument was also configured for other high‐resolution isotopologue applications, including CO_2_ and N_2_O clumped‐isotope measurements and SO_2_F_2_‐based sulfate clumped‐isotope analysis. Because the instrument has two independent dual‐inlet systems, SO_2_F_2_ was introduced through one system, while methane and the other gas species were analyzed through the second. Periods of continuous methane clumped‐isotope operation were defined as individual analytical campaigns and are summarized in Table S1.

Bulk carbon and hydrogen isotope compositions are expressed in conventional δ notation relative to VPDB and VSMOW, respectively:
(1)

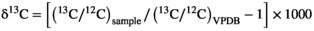

(2) $$\mathrm{\delta D}=\left[{\left(D/H\right)}_{\text{sample}}/{\left(D/H\right)}_{\text{VSMOW}}-1\right]\times 1000$$

where δ^13^C and δD values are reported in ‰. The bulk isotope measurements were calibrated to the VPDB and VSMOW scales using the shared CH₄ standard AL1 from MIT [5], following the procedure described by Zhang et al. [9]. The clumped‐isotope compositions are expressed as Δ^13^CH₃D and Δ^12^CH_2_D_2_ in ‰, representing the deviations of the measured ^13^CH₃D/^12^CH₄ and ^12^CH_2_D_2_/^12^CH₄ isotopologue ratios, respectively, from those expected for a stochastic distribution of isotopes:
(3)

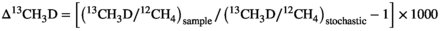



(4)

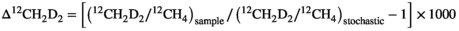




The corresponding stochastic isotopologue abundances are calculated from the bulk carbon and hydrogen isotope compositions of each sample as described previously [7, 9].

The analytical procedure followed Zhang et al. [9], with δ^13^C, δD, Δ^13^CH₃D, and Δ^12^CH_2_D_2_ determined using three sequentially operated measurement configurations implemented in Qtegra. For simultaneous δ^13^C and Δ^12^CH_2_D_2_ analysis, the instrument was operated in high‐resolution (HR) mode at a mass‐resolving power (MRP) of 28 000–33 000. The ^12^CH₄^+^, ^13^CH₄^+^, and ^12^CH_2_D_2_^+^ ion beams were collected using the L4 and L1 Faraday cups and the H4 CDD, respectively. This measurement required approximately 14–15 h. Contributions of the adjacent ^13^CH₃D^+^ and ^13^CH₅^+^ peaks to the ^12^CH_2_D_2_^+^ signal were corrected using their measured intensities and tailing factors derived from an independently measured water‐peak shape, following Eldridge et al. [8] and Zhang et al. [9].

Hydrogen isotope ratios were subsequently measured in HR + mode at a MRP of 45 000–50 000, allowing separation of the ^12^CH₃D^+^ peak from neighboring adduct peaks. The ^12^CH₃D^+^ and ^12^CH₄^+^ signals were recorded using the H4 CDD and L1 Faraday cup, respectively, over approximately 4 h. Finally, Δ^13^CH₃D was measured in HR mode at a MRP of 20 000–25 000. The ^13^CH₃D^+^ beam was measured on the H3 CDD at a flat position on the peak shoulder, while ^12^CH₄^+^ was monitored simultaneously using the L4 Faraday cup. The analytical position was checked manually at regular intervals to account for peak drift, and each Δ^13^CH₃D measurement required approximately 5 h.

A new narrow entrance slit was generally installed before each methane campaign to ensure sufficient ion transmission at high mass resolution. Slit condition was monitored using the ratio of the ^12^CH₄^+^ signal measured in medium resolution (MR) and HR modes with the L4 Faraday cup. A newly installed slit typically yielded an MR/HR intensity ratio of ~3. The slit was generally replaced when this ratio increased above 7, indicating substantially reduced ion transmission in HR mode, at which point the low‐abundance ^12^CH_2_D_2_^+^ beam was insufficient to maintain an internal precision better than 2‰. Further details of the collector configurations, measurement cycles, and background and tailing corrections are provided in Zhang et al. [9].

Source‐tuning parameters were optimized empirically for each analytical campaign to maximize ion intensity while maintaining suitable peak shape and resolution, rather than being kept at fixed settings throughout the study. In practice, Focus Quad II was particularly important for resolution and peak shape, while other source and focusing parameters could also influence tailing and were adjusted iteratively when necessary. The emission control current was fixed at 1.8 mA throughout the methane analyses.

### 2.2 Experimental Design

#### 2.2.1 Zero Tests and Working Gas Monitoring From 2019 to 2026

At the beginning of each methane analytical campaign, the ion source and electrostatic analyzer (ESA), together with the dual‐inlet bellows and capillaries, were baked for at least 8 h, followed by a cooling and stabilization period of around 24 h. An RG, designated STD‐B (δ^13^C_VPDB_ = −38.45‰, δD_VSMOW_ = −168.60‰, Δ^13^CH₃D = +2.50‰, and Δ^12^CH_2_D_2_ = +3.57‰), and a laboratory working gas (WG), designated STD‐C (δ^13^C_VPDB_ = −47.36‰, δD_VSMOW_ = −173.54‰, Δ^13^CH₃D = +2.44‰, and Δ^12^CH_2_D_2_ = +2.54‰), which served as an independent quality‐control gas, were routinely analyzed before and between sample measurements (Table S2). Both RG and WG were high‐purity commercial methane gases (> 99.9%). To isolate instrumental variability from potential errors or isotope offsets introduced during sample preparation, all RG and WG measurements considered here were performed by direct introduction without pretreatment such as purification. The bulk and clumped‐isotope compositions of both gases were established using a series of Ni‐catalyzed equilibrated methane gases measured during 2020 [9].

Zero tests were performed by introducing the same RG into both the sample and reference bellows and measuring it under identical analytical conditions. The resulting deviations from zero were used to evaluate the short‐term stability and repeatability of the measurement system under nominally identical sample–reference conditions. Measurements of WG against RG were used as an independent long‐term check of analytical reproducibility and calibration stability. Repeated WG analyses allowed us to assess whether instrumental drift or changes in instrumental configuration introduced systematic offsets relative to the calibration framework established during 2020, and whether this framework remained applicable to measurements conducted from 2021 to 2026.

Between methane analytical campaigns, the instrument was frequently reconfigured for other high‐resolution isotopologue applications, including SO_2_F_2_‐based sulfate clumped‐isotope analysis, and underwent routine maintenance or component replacement. These interventions included bakeout, entrance‐slit replacement, ion‐source tuning and cleaning, ion‐source or filament replacement, CDD replacement, replacement of dual‐inlet components, and replacement of the HV supply module. The combined records of zero tests and repeated WG measurements were therefore used to evaluate whether these operational changes affected instrumental stability and the continued validity of the original calibration framework.

#### 2.2.2 Equilibrated Methane Gases Prepared From 2020 to 2026

Equilibrated methane gases were prepared and measured both to establish the original calibration framework and to evaluate its subsequent validity. The gases prepared in 2020 were used to define the calibration framework reported by Zhang et al. [9], whereas additional gases prepared and measured from 2021 to 2026 served as independent postcalibration checks. The theoretical equilibrium Δ^13^CH₃D and Δ^12^CH_2_D_2_ values at each equilibration temperature were calculated using the temperature‐dependent relationships reported by Young et al. [7]. The preparation conditions and analytical dates of individual gases, including equilibration temperature and duration, are summarized in Table S3.

From 2020 to 2022, equilibrated methane gases were prepared at Science Tokyo using Ni as the catalyst at temperatures between 300°C and 500°C. Methane was sealed in glass tubes or vials and heated in temperature‐controlled ovens or heating units, following the procedures described by Zhang et al. [9]. After equilibration, the gases were purified using a GC‐4000 gas chromatograph (GL Sciences) equipped with two Hayesep Q columns maintained at 30°C.

From 2024 to 2026, additional equilibrated methane gases were prepared at Empa using γ‐Al_2_O₃ as the catalyst in sealed stainless‐steel vessels at temperatures up to 300°C, following Zhang et al. [10]. Under these conditions, γ‐Al_2_O₃‐catalyzed equilibration did not measurably generate impurity gases or alter the original bulk isotopic composition. High‐purity methane gases, including EP4 and EP6, were therefore introduced directly without further purification. Source gases containing substantial nonmethane components, including BIO and M2, were purified using a cryogen‐aided gas chromatograph equipped with two sequential Carboxen 1000 columns at ETH Zurich. This protocol allows quantitative separation of methane from the other gas components without measurable isotopic fractionation within analytical uncertainty [25].

Two subsets of equilibrated gases provided controlled tests of possible δD‐dependent bias while minimizing covariance with δ^13^C (Table S3): (i) Ni‐catalyzed gases equilibrated at 335°C during 2021–2022, with δ^13^C values of −25.4‰ ± 0.5‰ and δD values ranging from approximately −282‰ to −10‰; and (ii) four γ‐Al_2_O₃‐catalyzed gases equilibrated at 220°C in 2026, with δ^13^C values of −44.0‰ ± 0.9‰ and δD values ranging from −195‰ to −50‰. Together with the remaining equilibrated gases, they provide a long‐term dataset for assessing both the continued validity of the original calibration framework and the possible need for additional bulk‐isotope–dependent corrections.

#### 2.2.3 Interlaboratory Comparison Dataset

An interlaboratory comparison dataset was compiled to evaluate the external consistency of the Science Tokyo 253 Ultra calibration framework. The dataset comprises previously reported measurements and additional unpublished Science Tokyo measurements of nine shared methane gases, with measurements obtained across seven laboratories using three analytical platforms: 253 Ultra HR‐IRMS, Panorama HR‐IRMS, and mid‐infrared laser spectroscopy (Table S5). The laboratories represented in the dataset are the Institute of Science Tokyo (Science Tokyo), Tianjin University (TJU), the Guangzhou Institute of Geochemistry, Chinese Academy of Sciences (GIGCAS), Utrecht University (UU), the Massachusetts Institute of Technology (MIT), the University of California, Los Angeles (UCLA), and the Swiss Federal Laboratories for Materials Science and Technology (Empa).

STD‐A and STD‐C were obtained from commercial methane cylinders used as laboratory gases at Science Tokyo and their calibrated bulk‐ and clumped‐isotope compositions were reported by Zhang et al. [9]. Clumped‐isotope measurements of STD‐A and STD‐C were also reported by TJU using the 253 Ultra [11] and by GIGCAS using the Panorama [15]. The methane gases shared by MIT include two commercial laboratory RGs, AL1 and AS2, and two isotopically modified mixtures, AL1‐CD and AL1‐D3. The latter were prepared to produce ^13^C‐depleted and D‐enriched methane compositions, respectively. Measurements of these gases by laser spectroscopy and Panorama HR‐IRMS were previously reported by Gonzalez et al. [5] and Young et al. [6]. EP1, EP6, and EP7 are commercial methane gases shared by Empa. These gases were measured at Science Tokyo and UU using the 253 Ultra and at Empa using laser spectroscopy, as reported by Zhang et al. [10].

The Science Tokyo measurements of STD‐A, STD‐C, AL1, AS2, AL1‐CD, and AL1‐D3 were obtained during the 2020–2021 analytical campaigns, whereas EP1, EP6, and EP7 were measured in 2024.

## 3 Results and Discussion

### 3.1 Long‐Term Reproducibility of Reference and Working Gases

Figure 1 summarizes the long‐term variability of RG zero tests and WG measurements conducted during methane analytical campaigns from 2019 to 2026. For the RG zero tests, residuals represent the measured Δ values from RG versus RG analyses, whereas for the WG measurements, they are the differences between the measured and assigned WG Δ values reported in Zhang et al. [9].

**FIGURE 1:**
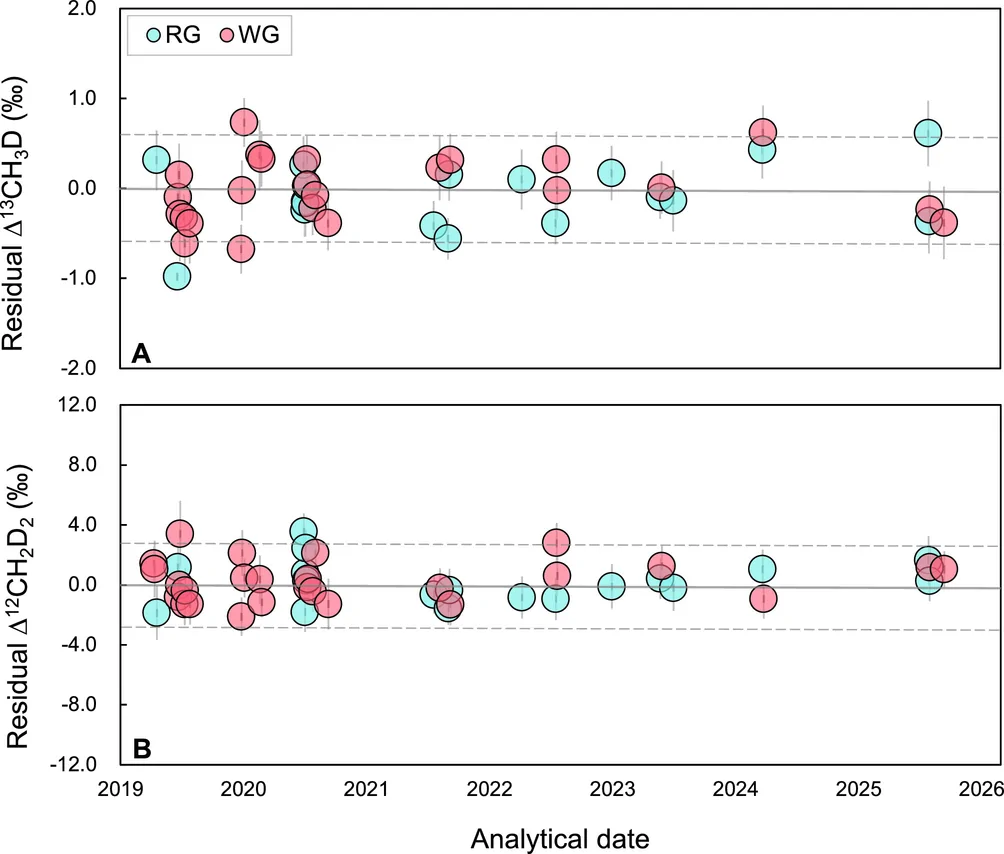
Reproducibility of RG and WG measurements during CH₄ analytical campaigns from 2019 to 2026. Residuals are calculated relative to expected values defined by the 2020 equilibrated CH₄ gas calibration [9] for (A) Δ^13^CH₃D and (B) Δ ^12^CH_2_D_2_. The solid horizontal lines indicate zero residual, whereas the dashed lines indicate ±2*σ* external reproducibility based on the long‐term analytical precision previously established for the Science Tokyo 253 Ultra: ±0.6‰ for Δ^13^CH₃D and ±3‰ for Δ ^12^CH_2_D_2_ [9]. Error bars represent ±1 standard error.

Repeated RG zero tests and WG measurements show that the analytical system maintained stable long‐term reproducibility across multiple methane clumped‐isotope campaigns. Residuals of both Δ^13^CH₃D and Δ^12^CH_2_D_2_ were generally centered around zero and mostly remained within the expected ±2*σ* external reproducibility limits. Although several individual measurements fell outside these limits, these deviations were isolated and did not persist across consecutive campaigns or show a systematic temporal trend.

These two tests provide complementary constraints on long‐term performance of 253 Ultra. RG zero tests assess measurement stability under nominally identical sample–reference conditions, whereas WG measurements test whether a gas with independently assigned clumped‐isotope values continued to reproduce its expected composition relative to the RG. Their consistent behavior indicates that measurements from different analytical campaigns can be compared within the expected external reproducibility, without applying campaign‐specific offset corrections. Together with the equilibrated‐gas results discussed below, these reference‐gas checks indicate that the original calibration framework remained applicable to subsequent measurements.

### 3.2 Persistence of the 2020 Calibration Framework

Equilibrated methane gases provide a more direct test of calibration persistence than repeated WG measurements alone. The 2020 Ni‐equilibrated gases were used to establish the original calibration framework, whereas independently prepared gases measured from 2021 to 2026 served as postcalibration checks. For each gas, the residual was calculated as the measured Δ value minus the theoretical equilibrium value predicted for its equilibration temperature.

As shown in Figure 2, postcalibration residuals remained generally centered near zero for both Δ^13^CH₃D and Δ^12^CH_2_D_2_, with no systematic temporal trend from 2021 to 2026. Most measurements remained within the expected ±2*σ* external reproducibility limits for 253 Ultra methane clumped‐isotope measurements. Several individual residuals approached or exceeded these limits, but these deviations occurred in both positive and negative directions and were not reproduced in subsequent measurements or campaigns. This pattern indicates analytical variability rather than systematic calibration drift.

**FIGURE 2:**
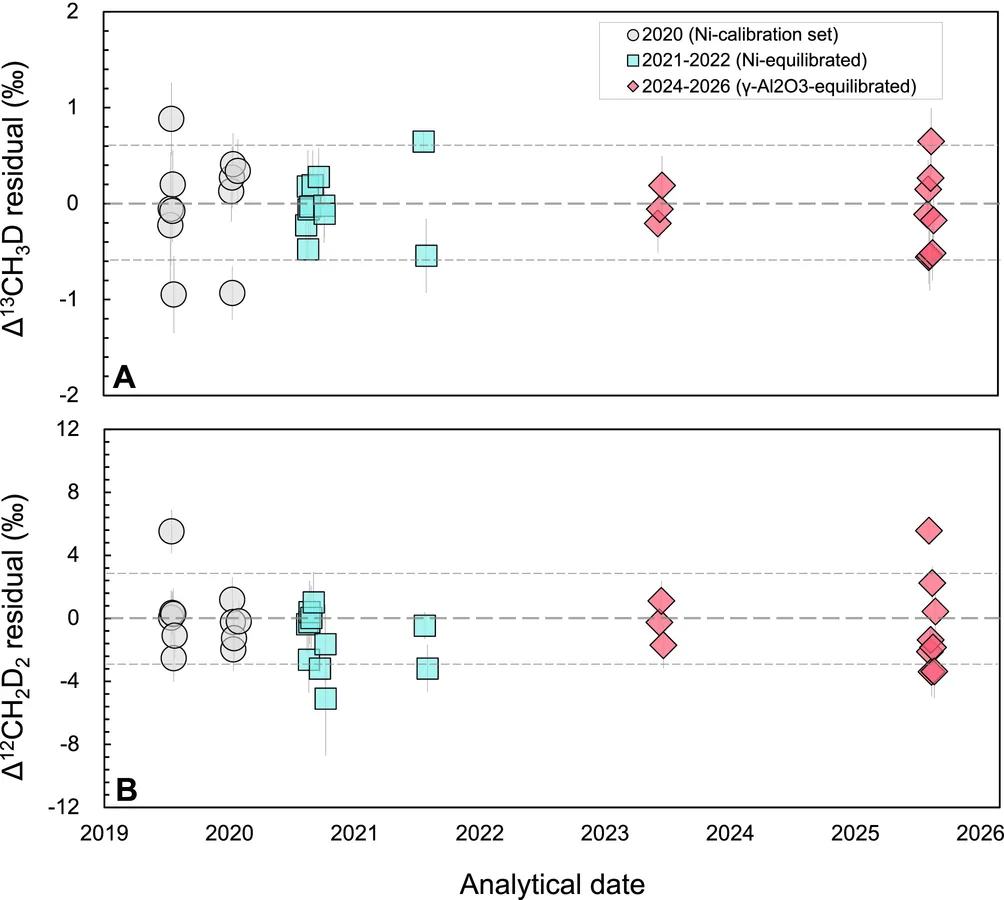
Clumped isotope residuals of equilibrated gases analyzed from 2020 to 2026. Residuals are calculated as measured values minus the theoretical equilibrium values at the corresponding equilibration temperatures [7] for (A) Δ^13^CH₃D and (B) Δ ^12^CH_2_D_2_. The 2020 Ni‐equilibrated gases were used to establish the original calibration framework for reference‐gas and sample measurements [9] and are shown for comparison as light‐grey circles. The 2021–2026 gases, shown as blue squares and pink diamonds, provide postcalibration checks of the persistence of this framework in subsequent analytical campaigns. Horizontal dashed lines indicate zero residual and the ±2*σ* external reproducibility. Error bars represent ±1 standard error.

The measured and predicted equilibrium values are compared directly in Figure S1. The gases were equilibrated at temperatures from 220°C to 500°C, providing multiple independently defined equilibrium compositions in addition to the fixed RG and WG compositions. Data from the 2021–2026 campaigns continued to plot around the 1:1 relationship defined by the original calibration gases, with no evidence of a campaign‐dependent offset or systematic change in calibration response.

These results show that the calibration framework established in 2020 remained applicable to equilibrated gases prepared and measured in later campaigns. This agreement is notable because the gases were prepared in different laboratories using different catalysts, equilibration systems, and purification procedures, whereas their expected clumped‐isotope compositions were defined by equilibration temperature rather than by assigned laboratory values, as was the case for the WG discussed in Sect. 3.1.

### 3.3 Bulk‐Isotope Dependence of Calibrated Clumped‐Isotope Values

Ideally, calibrated clumped‐isotope values should be independent of bulk isotopic composition. In practice, however, incomplete corrections or instrument‐specific analytical effects may introduce apparent bulk‐isotope dependence. Some laboratories have reported such effects for Panorama HR‐IRMS measurements [15], while others found them smaller than analytical precision [6, 14]. These contrasting observations suggest that bulk‐isotope–dependent behavior may vary among individual instruments and operating conditions and therefore may need to be evaluated separately for 253 Ultra measurements. To date, Eldridge et al. [8] reported no significant δD dependence over a range of −160‰ to +20‰ for a 253 Ultra, but whether the same conclusion applies to the much lower δD values characteristic of microbial methane remains uncertain. We therefore examined whether residuals between measured and predicted equilibrium values varied systematically with δD or δ^13^C, extending the δD range to about −335‰.

The most direct test of δD dependence is provided by two subsets of equilibrated gases in which δ^13^C remained relatively constant while δD varied over a wide range (Figure S2). The Ni‐equilibrated gases measured during 2021–2022 had δ^13^C values of −25.4‰ ± 0.5‰ and δD values ranging from −282‰ to −10‰. A second subset of γ‐Al_2_O₃‐equilibrated gases had δ^13^C values of −44.0‰ ± 0.9‰ and δD values ranging from −195‰ to −50‰. Neither Δ^13^CH₃D nor Δ^12^CH_2_D_2_ residuals show an apparent systematic variation with δD within either subset (Figure S2), providing no evidence that an additional empirical δD correction is required over the investigated δD ranges.

Extending this analysis from the restricted δ^13^C subsets in Figure S2 to the full equilibrated‐gas dataset yields the same general conclusion (Figure 3). Residuals for both clumped‐isotope values remain distributed around zero across δD values from −335‰ to −10‰ (Figure 3B,D). In particular, the lowest‐δD gases do not show systematically higher or lower residuals than gases with thermogenic‐like δD values. No systematic relationship is evident between residuals and δ^13^C over the investigated range (Figure 3A,C). Although the δ^13^C comparison is less tightly controlled, the absence of a consistent trend across campaigns suggests that any δ^13^C‐dependent effect, if present, is small relative to the external reproducibility of the method.

**FIGURE 3:**
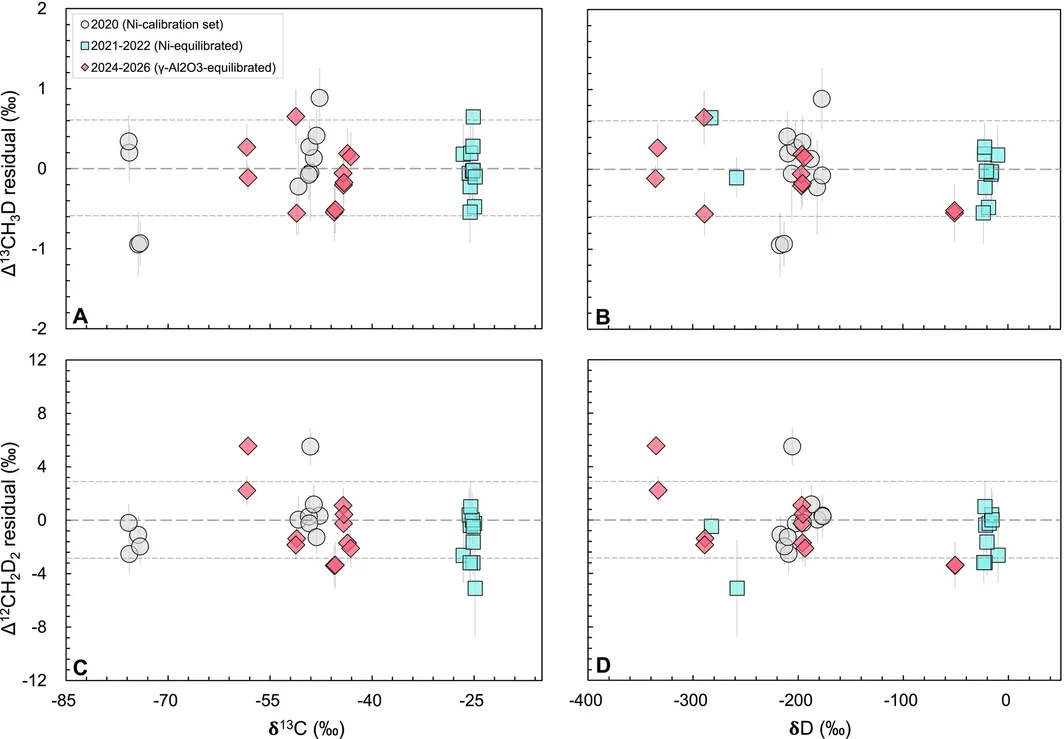
Clumped‐isotope residuals of equilibrated gases plotted against bulk isotopic compositions: (A) Δ^13^CH₃D versus δ ^13^C; (B) Δ^13^CH₃D versus δD; (C) Δ ^12^CH_2_D_2_ versus δ^13^C; and (D) Δ^12^CH_2_D_2_ versus δD. Symbols and horizontal lines are as defined in Figure 2. Error bars represent ±1 standard error.

Although no empirical δD dependence was resolved in this study, low‐δD methane remains analytically more sensitive to peak‐tailing effects in Δ^12^CH_2_D_2_ measurements. Under typical analytical conditions, the ^12^CH_2_D_2_^+^ signal was on the order of 80 cps for the RG (δD = −168.60‰), compared with ~50 cps for microbial methane with δD ≈ −335‰. An identical absolute residual tailing contribution therefore represents a larger fraction of the minor‐isotopologue signal in the low‐δD sample and can produce a greater sample–reference bias if it is not accurately corrected.

To illustrate this effect over a broader compositional range, the apparent Δ^12^CH_2_D_2_ bias was calculated as a function of sample δD for residual tailing errors of 0.5–2.0 cps (Figure 4). To isolate the effect of bulk δD, these calculations assume a constant true Δ^12^CH_2_D_2_ value for the sample and RG, such that changes in the predicted ^12^CH_2_D_2_^+^ signal arise only from differences in δD. The calculated bias is negligible when the sample δD is close to that of the RG but increases progressively as the sample becomes more D‐depleted. At δD ≈ −335‰, residual tailing errors of 1 and 2 cps produce apparent Δ^12^CH_2_D_2_ shifts of approximately 7‰ and 14‰, respectively. To evaluate the sensitivity under actual analytical conditions, the same calculation was also applied using measured signal intensities for STD‐C, BIO, and BIO‐HG (Figure 4 and Table S4). For BIO, a microbial methane sample with δD = −336.7‰ and Δ^12^CH_2_D_2_ = −40.8‰, the measured ^12^CH_2_D_2_^+^ signal was 44.7 cps, corresponding to estimated biases of 4.3‰–17.0‰ for residual errors of 0.5–2.0 cps. BIO‐HG, prepared by equilibrating methane from the same parent gas at 220°C, had a similar δD (−335.1‰) but a higher Δ^12^CH_2_D_2_ value (+10.3‰) and ^12^CH_2_D_2_^+^ signal (50.1 cps), giving estimated biases of 3.5–13.8‰. In comparison, STD‐C, with a δD value close to that of the RG, showed corresponding biases of only 0.1‰–0.5‰. Thus, the strongly negative Δ^12^CH_2_D_2_ value of microbial methane further increases its sensitivity to residual tailing, although this additional effect is relatively small compared with the overall sensitivity associated with its low δD.

**FIGURE 4:**
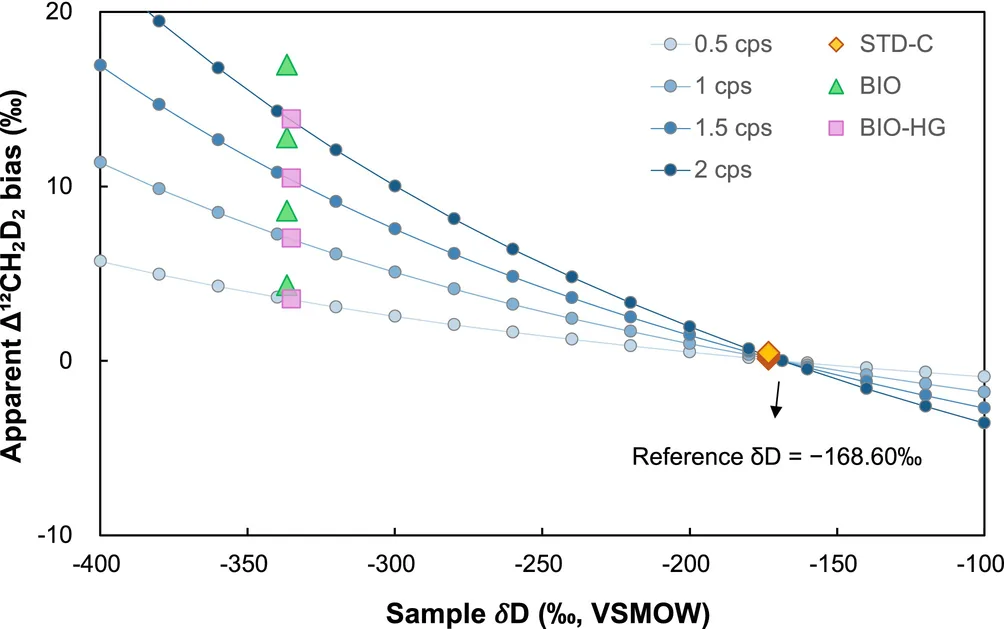
Sensitivity of apparent Δ^12^CH_2_D_2_ values to residual peak‐tailing errors as a function of sample δD. Calculations assume a reference gas with δD = −168.60‰ and a ^12^CH_2_D_2_^+^ signal of 80 cps. To isolate the effect of bulk δD, sample ^12^CH_2_D_2_^+^ intensities were calculated assuming a constant true Δ^12^CH_2_D_2_ value for the sample and reference gas. Curves show the apparent Δ^12^CH_2_D_2_ bias resulting from positive residual tailing errors of 0.5, 1.0, 1.5, and 2.0 cps remaining in both sample and reference signals after incomplete tailing correction. Overcorrection would result in biases of opposite sign. Diamonds, triangles, and squares show sensitivity estimates based on measured signal intensities for STD‐C, BIO, and BIO‐HG, respectively, with the four points for each sample corresponding, from bottom to top, to residual tailing errors of 0.5, 1.0, 1.5, and 2.0 cps. BIO is a microbial methane sample, whereas BIO‐HG was prepared by equilibrating methane from the same parent gas at 220°C. Corresponding measured signal intensities and calculated biases are provided in Table S4.

This behavior illustrates why measurements of gases isotopically similar to the RG are relatively insensitive to residual peak‐tailing effects, whereas low‐δD methane provides a more stringent test of the analytical and calibration procedures. Accordingly, repeated measurements of equilibrated gases spanning a wide δD range can reveal residual bulk‐isotope–dependent biases arising from incomplete peak‐tailing correction or other instrument‐specific analytical effects. For the 253 Ultra analytical protocol used at Science Tokyo, the absence of a systematic residual trend with decreasing δD in the equilibrated gases, down to −335‰, indicates that the applied corrections remained effective over the range examined.

Overall, the equilibrated‐gas results extend the δD range previously evaluated for the 253 Ultra and provide no evidence for a bulk‐isotope–dependent bias requiring an additional empirical correction for either Δ^13^CH₃D or Δ^12^CH_2_D_2_.

### 3.4 External Validation Using Shared Methane Gases

Measurements of methane gases shared with other laboratories provide an external test of the Science Tokyo 253 Ultra calibration framework. Because the Science Tokyo results were obtained during the 2020‐C1/C2, 2021‐C1, and 2024‐C1 analytical campaigns, the comparison also assesses the persistence of the calibration framework over multiple years.

Overall, the Science Tokyo values are consistent with measurements from other laboratories for most shared gases (Table S5 and Figure 5). This agreement is maintained over a wide range of bulk isotopic compositions, from −81.9‰ to −34.5‰ in δ^13^C and from −211.3‰ to +243.4‰ in δD, indicating that the Science Tokyo calibration framework is not limited to methane gases with thermogenic‐like bulk compositions. The shared gases also test calibration transferability for methane with nonequilibrium clumped‐isotope compositions. STD‐A and AL1‐CD represent gases with elevated Δ^13^CH₃D values of 45.6‰ and 15.6‰, respectively, whereas AL1‐D3 represents a gas with positive Δ^12^CH_2_D_2_ of 29.6‰. Their consistency with other laboratories within, or close to, reported uncertainties indicates that the Science Tokyo calibration framework is applicable beyond methane at or near thermal equilibrium. Because these gases were analyzed in different campaigns from 2020 to 2024, the comparison further supports the long‐term stability inferred from the internal reference‐gas monitoring and equilibrated‐gas validation discussed above.

**FIGURE 5:**
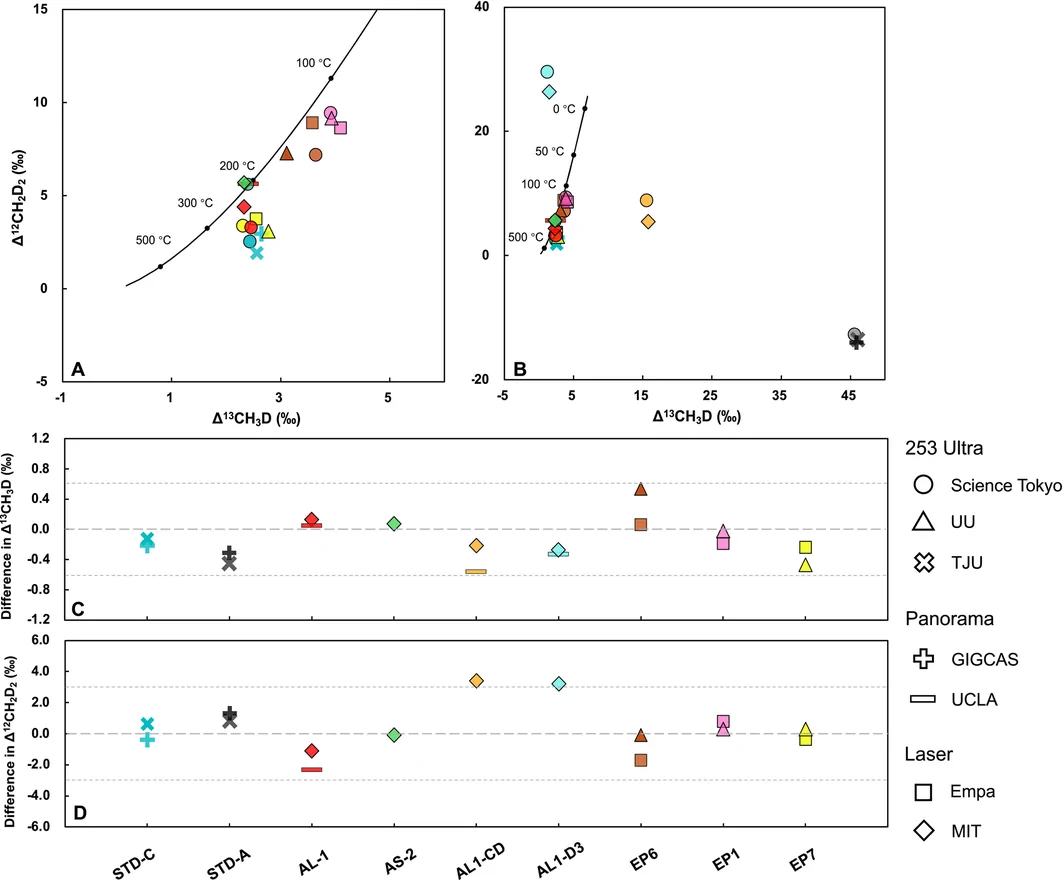
Comparison of methane clumped‐isotope measurements across laboratories. (A) Δ^13^CH₃D and (B) Δ^12^CH_2_D_2_ values measured for different methane samples, shown together with the theoretical equilibrium relationship (black curve); (C) differences in Δ^13^CH₃D and (D) differences in Δ^12^CH_2_D_2_, calculated as the Science Tokyo measurement minus the corresponding value obtained by each of the other laboratories. The dashed lines in panels (C) and (D) indicate the representative ±2*σ* external reproducibility defined in Figure 1, which is broadly comparable to the analytical precision reported for the different platforms. The complete dataset is provided in Table S5. Colors consistently identify individual samples across all panels, whereas symbols denote laboratories and analytical platforms.

The present comparison also defines the main gaps for future interlaboratory calibration. First, this dataset does not include very low‐δD microbial methane. Such samples have low ^12^CH_2_D_2_ abundance and are therefore more sensitive to peak tailing and background correction in 253 Ultra measurements; for laser spectroscopy, they may also place stronger demands on spectral fitting and baseline treatment [10]. Second, more shared standards with strongly disequilibrated Δ^12^CH_2_D_2_ values are needed. Natural methane can span a much wider Δ^12^CH_2_D_2_ range, from values approaching −60‰ in microbial methane [14, 26, 27] to values as high as +50‰ in atmospheric methane [14, 28]. Addressing these gaps will require coordinated community efforts to prepare and distribute shared methane standards that cover both low‐δD microbial‐like compositions and strongly disequilibrated Δ^12^CH_2_D_2_ compositions, allowing laboratories using different instruments to validate measurement and calibration strategies and minimize platform‐dependent discrepancies.

## 4 Conclusions

Long‐term monitoring of methane clumped‐isotope measurements at Science Tokyo demonstrates that the 253 Ultra can provide stable Δ^13^CH₃D and Δ^12^CH_2_D_2_ data over multiyear operation. This stability is supported by reproducible zero‐test and working‐gas measurements, along with independent postcalibration equilibrated‐gas checks. External consistency is further supported by interlaboratory comparison of shared methane standards. Together, these results show that 253 Ultra methane clumped‐isotope measurements can remain stable over long‐term operation without requiring a new equilibrium calibration, provided that reference‐gas behavior, working‐gas reproducibility, and equilibrated‐gas checks remain consistent.

Possible bulk‐isotope dependence was evaluated using equilibrated methane gases, for which Δ^13^CH₃D and Δ^12^CH_2_D_2_ values are constrained by the equilibration temperature. Residuals between measured and temperature‐predicted values show no systematic dependence on bulk δ^13^C or δD over the ranges examined, including gases with δD values as low as −335‰. These results indicate that no additional empirical bulk‐isotope correction is required under the Science Tokyo analytical conditions. Low‐δD methane nevertheless requires careful tailing and background correction because of its lower ^12^CH_2_D_2_ abundance.

More broadly, this study provides a practical framework for other laboratories using 253 Ultra instruments to evaluate long‐term stability and possible bulk‐isotope–dependent biases in methane clumped‐isotope measurements. Repeated zero tests, working‐gas monitoring, the analysis of independently equilibrated methane gases, and shared‐gas comparisons together offer complementary checks on instrumental stability, calibration persistence, and interlaboratory compatibility. Future interlaboratory efforts should prioritize shared standards representing very low‐δD microbial methane and strongly disequilibrated Δ^12^CH_2_D_2_ compositions, which remain insufficiently covered by existing comparison datasets.

## Author Contributions

**Naizhong Zhang:** conceptualization, methodology, investigation, validation, formal analysis, funding acquisition, visualization, data curation, writing – review and editing, writing – original draft. **Alexis Gilbert:** funding acquisition, writing – review and editing, supervision. **Mayuko Nakagawa:** methodology, investigation, writing – review and editing. **Jakub Surma:** methodology, writing – review and editing. **Jan G. C. Meissner:** investigation, writing – review and editing. **Joachim Mohn:** funding acquisition, supervision, writing – review and editing, resources. **Stefano M. Bernasconi:** funding acquisition, supervision, writing – review and editing, resources. **Yuichiro Ueno:** resources, writing – review and editing, supervision. **Naohiro Yoshida:** supervision, resources, writing – review and editing.

## Funding

This work was supported by the Japan Society for the Promotion of Science (20K14594, 22K14134, and 22H05151) and the Schweizerischer Nationalfonds zur Förderung der Wissenschaftlichen Forschung (200021_200977).

## Supporting information


**S1:** Sensitivity calculation for residual peak‐tailing effects.
**Figure S1:** Measured versus expected values of (A) Δ^13^CH₃D and (B) Δ^12^CH_2_D_2_ for equilibrated gases analyzed during different CH₄ analytical campaigns. The thick dashed line indicates the 1:1 relationship, whereas the thin dashed lines indicate the ±2*σ* external reproducibility envelope for each isotopologue. Campaign IDs are defined in Table S1. The 2020‐C1 and 2020‐C2 gases were used to establish the original calibration framework and are included for comparison. The distribution of the postcalibration gases around the 1:1 line provides a test for campaign‐dependent offsets or changes in calibration response across the measured equilibrium range.
**Figure S2:** Evaluation of δD dependence in clumped isotope measurements using equilibrated gases with restricted δ^13^C ranges. The 2021–2022 dataset (blue squares) was equilibrated using Ni as the catalyst and has δ^13^C = −25.4‰ ± 0.5‰, whereas the 2024–2026 dataset (pink diamonds) was equilibrated using γ‐Al_2_O₃ and has δ^13^C = −44.0‰ ± 0.9‰. Residuals are calculated as described in Figure 2.

**Table S1:** CH₄ analytical campaigns on the Science Tokyo 253 Ultra.
**Table S2:** Long‐term zero‐test and working‐gas datasets for STD‐B (RG) and STD‐C (WG) measured on the Science Tokyo 253 Ultra.
**Table S3:** Preparation conditions and measured clumped‐isotope compositions of equilibrated methane gases prepared from 2020 to 2026.
**Table S4:** Measured signal intensities and estimated apparent Δ^12^CH_2_D_2_ biases for representative methane samples used in the sensitivity analysis.
**Table S5:** Interlaboratory comparison of shared methane gases measured using 253 Ultra, Panorama, and laser‐based platforms.

## Data Availability

The data that supports the findings of this study are available in the supplementary material of this article.
