# Supplementary material for: Long‐Term Performance and Bulk‐Isotope Effects in Methane Clumped‐Isotope Measurements Using a 253 Ultra HR‐IRMS
Source: Rapid Commun Mass Spectrom. 2026 Oct 23;41(1):e70198. doi: 10.1002/rcm.70198 (PMC13693374; doi:10.1002/rcm.70198)
Supplement: Supplementary file 1 [file RCM-41-e70198-s001.docx]

**Supporting Information**

**Long-term performance and bulk-isotope effects in methane clumped-isotope measurements using a 253 Ultra HR-IRMS**

Naizhong Zhang et al.

**S1. Sensitivity calculation for residual peak-tailing effects**

The sensitivity of calculated Δ¹²CH₂D₂ values to residual peak-tailing errors was evaluated using a simplified isotope-abundance model. The calculation was designed to isolate the effect of bulk δD differences between sample and reference gas, while keeping the true Δ¹²CH₂D₂ composition constant.

A reference gas (STD-B) with δD = −168.60‰ and a ¹²CH₂D₂⁺ signal intensity of 80 cps, representative of typical analytical conditions at Science Tokyo, was used as the baseline condition. For a sample with a different δD value, the expected ¹²CH₂D₂⁺ signal intensity was estimated assuming that the abundance of ¹²CH₂D₂ scales with the square of the D/H ratio. The sample signal was therefore calculated as

I_18_s_ = I_18_r_ * [(1+ δD_s_/1000)/(1+δD_r_/1000)]^2^

where I_18_s_ and I_18_r_ are the ¹²CH₂D₂⁺ signal intensities of the sample and reference gas, respectively, and δD_s_ and δD_r_ are their bulk hydrogen-isotope compositions.

The effect of incomplete peak-tailing correction was then simulated by assuming that the same residual tailing error, “a”, remained in both sample and reference ¹²CH₂D₂⁺ signals after correction. The apparent sample-to-reference ratio becomes

R’ = (I_18_s_ + a)/(I_18_r_ + a)

whereas the corresponding ratio in the absence of residual tailing is

R_true_ = I_18_s_/I_18_r_

Because Δ¹²CH₂D₂ is expressed as a relative deviation in an isotopologue ratio, the bias introduced by residual tailing was calculated from the fractional change in the sample-to-reference ratio, R’/R_true_ – 1, and converted to per mil units by multiplying by 1000. The resulting apparent bias in Δ¹²CH₂D₂ was then calculated as

Δ^12^CH_2_D_2_bias_ = (R’/R_true_ – 1) * 1000 = {[( I_18_s_ + a) /(I_18_r_ + a)] / (I_18_s_/I_18_r_) – 1} * 1000

Residual tailing errors of 0.5, 1.0, 1.5, and 2.0 cps were evaluated over a range of sample δD values. This range was selected as a conservative sensitivity range relative the magnitude and variability of peak-tailing corrections encountered during routine measurements. The magnitude of the correction can vary with instrument conditions and tuning but does not itself introduce a bias when accurately determined and applied. Because the absolute residual contribution represents an increasingly large fraction of the minor-isotopologue signal as δD decreases, the apparent Δ¹²CH₂D₂ bias increases progressively for samples that are more D-depleted than the reference gas. Positive residual errors represent incomplete tailing correction; over-correction would generate biases of similar magnitude but opposite sign.

This calculation is intended as a controlled sensitivity test rather than a prediction of the exact bias for individual natural methane samples. The exact magnitude of the resulting bias would also depend on the true Δ¹²CH₂D₂ composition, total ion transmission as affected by slit condition and instrument tuning, and differences in residual tailing between sample and reference gas.

To further evaluate the sensitivity under conditions representative of microbial methane, the same calculation was applied using measured ¹²CH₂D₂⁺ signal intensities from three representative measurements: STD-C, BIO, and BIO-HG. BIO and BIO-HG have similarly low δD values (−336.7‰ and −335.1‰, respectively), but substantially different Δ¹²CH₂D₂ values (−40.8‰ and +10.3‰, respectively). The measured signal intensities and resulting bias estimates are summarized in Table S4.

**Supporting Figures**

**Figure S1**. Measured versus expected values of (A) Δ¹³CH₃D and (B) Δ¹²CH₂D₂ for equilibrated gases analyzed during different CH₄ analytical campaigns. The thick dashed line indicates the 1:1 relationship, whereas the thin dashed lines indicate the ±2σ external reproducibility envelope for each isotopologue. Campaign IDs are defined in Table S1. The 2020-C1 and 2020-C2 gases were used to establish the original calibration framework and are included for comparison. The distribution of the post-calibration gases around the 1:1 line provides a test for campaign-dependent offsets or changes in calibration response across the measured equilibrium range.

**Figure S2**. Evaluation of δD dependence in clumped isotope measurements using equilibrated gases with restricted δ¹³C ranges. The 2021–2022 dataset (blue squares) was equilibrated using Ni as the catalyst and has δ¹³C = −25.4 ± 0.5‰, whereas the 2024–2026 dataset (pink diamonds) was equilibrated using γ-Al₂O₃ and has δ¹³C = −44.0 ± 0.9‰. Residuals are calculated as described in Figure 2.

**Supporting Tables**

Tables S1–S5 are provided in a separate Excel file.

**Table S1**. CH₄ analytical campaigns on the Science Tokyo 253 Ultra.

**Table S2**. Long-term zero-test and working-gas datasets for STD-B (RG) and STD-C (WG) measured on the Science Tokyo 253 Ultra.

**Table S3**. Preparation conditions and measured clumped-isotope compositions of equilibrated methane gases prepared from 2020 to 2026.

**Table S4**. Measured signal intensities and estimated apparent Δ¹²CH₂D₂ biases for representative methane samples used in the sensitivity analysis.

**Table S5**. Inter-laboratory comparison of shared methane gases measured using 253 Ultra, Panorama, and laser-based platforms.
